# Three Peptides from Soy Glycinin Modulate Glucose Metabolism in Human Hepatic HepG2 Cells

**DOI:** 10.3390/ijms161126029

**Published:** 2015-11-16

**Authors:** Carmen Lammi, Chiara Zanoni, Anna Arnoldi

**Affiliations:** Department of Pharmaceutical Sciences, University of Milan, Mangiagalli Street 25, 20133 Milan, Italy; carmen.lammi@unimi.it (C.L.); chiara.zanoni1@unimi.it (C.Z.)

**Keywords:** bioactive peptides, functional foods, glucose uptake, HepG2 cell line, plant proteins

## Abstract

Ile-Ala-Val-Pro-Gly-Glu-Val-Ala (IAVPGEVA), Ile-Ala-Val-Pro-Thr-Gly-Val-Ala (IAVPTGVA) and Leu-Pro-Tyr-Pro (LPYP), three peptides deriving from soy glycinin hydrolysis, are known to regulate cholesterol metabolism in human hepatic HepG2 cells. We have recently demonstrated that the mechanism of action involves the activation of adenosine monophosphate-activated protein kinase (AMPK). This fact suggested a potential activity of the same peptides on glucose metabolism that prompted us to also investigate this aspect in the same cells. After treatment with IAVPGEVA, IAVPTGVA and LPYP, HepG2 cells were analyzed using a combination of molecular techniques, including western blot analysis, glucose uptake experiments and fluorescence microscopy evaluation. The results showed that these peptides are indeed able to enhance the capacity of HepG2 cells to uptake glucose, via glucose transporter 1 GLUT1 and glucose transporter 4 GLUT4 activation, through the stimulation of protein kinase B Akt and adenosine monophosphate-activated protein kinase AMPK pathways, both involved in glucose metabolism.

## 1. Introduction

Soy foods provide useful health benefits [1,2,3], especially in the area of hypercholesterolemia prevention [4,5]. In the framework of a research aimed at assessing the role of proteins and peptides in this activity [6,7,8], we have recently characterized the molecular mechanism through which three peptides deriving from soy glycinin digestion with trypsin or pepsin, namely: Ile-Ala-Val-Pro-Gly-Glu-Val-Ala (IAVPGEVA), Ile-Ala-Val-Pro-Thr-Gly-Val-Ala (IAVPTGVA) and Leu-Pro-Tyr-Pro (LPYP) [9], modulate cholesterol metabolism in HepG2 cells [10]. These peptides were selected because preceding investigations by other Authors had shown that they inhibit *in vitro* the activity of 3-hydroxy-3-methylglutaryl CoA reductase (HMGCoAR) [9,11,12]. Our experiments have demonstrated that these peptides are able to increase the low density lipoprotein (LDL) receptor (LDLR) protein level, with the consequence of an enhanced capacity of HepG2 cells to uptake LDL [10]. In the same paper, we have also shown that the regulation of cholesterol metabolism involves the activation of adenosine monophosphate-activated protein kinase (AMPK), an observation that suggested that IAVPGEVA, IAVPTGVA and LPYP may also modulate glucose metabolism [10]. In fact, there is substantial evidence that AMPK is dysregulated in animal models and humans affected by the metabolic syndrome or type-2 diabetes and that the physiological or pharmacological activation of AMPK may improve insulin sensitivity and metabolic health [13]. AMPK activation leads to the inhibition of hepatic glucose production and stimulation of glucose uptake in hepatic cells, which helps to maintain the correct glycemia [14]. AMPK is therefore becoming an attractive target for type-2 diabetes therapies.

Regulation of glucose uptake from the blood and metabolism in peripheral tissues are key steps in maintaining a healthy metabolic phenotype. Glucose uptake into cells is facilitated and tightly controlled by glucose transporters that show diverse expressions among different tissues [15]. In general, a specific isoform, such as GLUT4, is activated in response to insulin through the activation of phosphatidylinositol-4,5-bisphosphate 3-kinase (PI3K)-protein kinase B (Akt) pathway. In response to insulin, the Akt activation, through phosphorylation at serine 473, leads to a translocation of GLUT4 on cellular membranes. Moreover, active Akt leads to an increase of glycogen synthase (GS) activity by phosphorylation/inhibition of glycogen synthase kinase 3 (GSK3). Active GS is then able to carry out the glycogen production from glucose.

HepG2 cells, which are a suitable cell model for studying certain function of human hepatocytes, show a blunted response to insulin and, in general, the glucose uptake is facilitated by GLUT1, which is highly expressed in the human hepatocytes and HepG2 cells as well [16]. Interestingly, the stimulation of AMPK activity is associated with enhancement of GLUT1-mediated glucose transport [17]. All these factors make the well-understood HepG2 model suitable for carrying out experiments to assess glucose uptake and metabolism [17].

Moreover, increasing evidences suggest that increased AMPK activity is associated with increased *GLUT4* gene expression without involvement of insulin [18]. Furthermore, treatment with the AMPK activator 5-aminoimidazole-4-carboxamide-1-β-d-ribonucleoside (AICAR) results in increased *GLUT4* gene expression in specific tissues, such as skeletal muscle [19], even though the underlying molecular mechanisms mediating this response are still unknown.

Interestingly, a few published studies provided evidence that soy peptides and/or proteins may exert a hypoglycemic activity either in animals [20,21] or in type-2 diabetic patients [22,23] and peptide mixtures obtained by pepsin-pancreatic hydrolysis of soy protein improve glucose uptake in muscle L6 cells [24].

Taking into account all these evidences, the objectives of the present investigation were twofold: (a) to verify whether IAVPGEVA, IAVPTGVA and LPYP are able to modulate glucose metabolism in HepG2 cells; (b) to accomplish a molecular characterization of the stimulated pathways.

## 2. Results and Discussion

**Figure 1:**
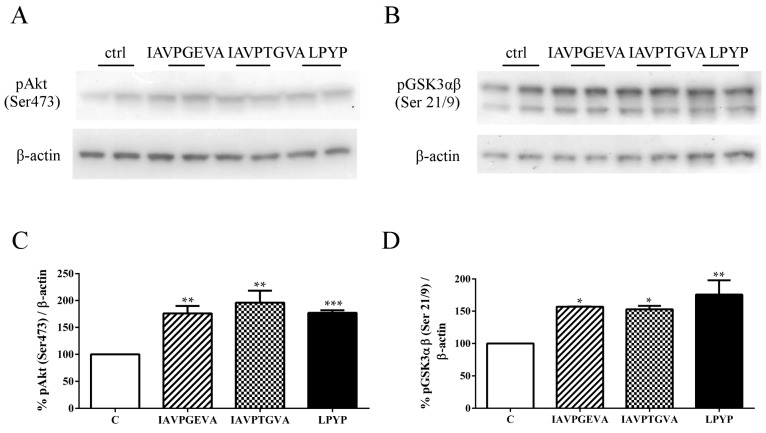
Effect of peptides on Akt/glycogen synthase kinase 3 αβ (Akt/GSK3αβ) pathway. HepG2 cells (1.5 × 10^5^) were treated with Ile-Ala-Val-Pro-Gly-Glu-Val-Ala (IAVPGEVA) (500 μM), Ile-Ala-Val-Pro-Thr-Gly-Val-Ala (IAVPTGVA) (500 μM) and Leu-Pro-Tyr-Pro (LPYP) (500 μM) for 24 h. (**A**,**B**) Exemplary western blot analyses of phosphorylated Akt (Ser473) and phosphorylated GSK3αβ (Ser21/9) levels after treatment. Signals were detected using specific anti-phospho-Akt (Ser473), anti-phospho-GSK3αβ (Ser21/9) and anti-β-actin primary antibodies (loading untreated sample); (**C**,**D**) Quantitative analyses of western blots. Phospho-Akt (Ser473) and phospho-GSK3αβ (Ser21/9) band intensities were quantified by ChemiDoc (BioRad, Hercules, CA, USA) and normalized using β-actin signals. Bars represent averages of duplicate samples ± SEM of six independent experiments. * *p* < 0.05, ** *p* < 0.001 *** *p* < 0.0001 *versus* untreated sample. C, untreated sample; pAkt, phosphor-Akt; pGSK3αβ, phospho-GSK3αβ.

In order to examine whether IAVPGEVA, IAVPTGVA and LPYP may affect the activation of Akt and GSK3αβ (its direct substrate and major target), western blot analyses were performed on lysates from treated HepG2 cells using antibodies specific for Akt phosphorylated at serine 473 and for GSK3αβ phosphorylated at serines 21 and 9, respectively. The results (Figure 1) suggest that these peptides activate the Akt pathway, since the treatments with IAVPGEVA, IAVPTGVA and LPYP significantly increased the level of phosphorylated Akt by 76%, 96% and 77%, respectively, *versus* the untreated sample. Akt activation determined in turn the inhibition of GSK3αβ activity by phosphorylation at (Ser 21/9) by 57%, 53% and 76%, respectively, *versus* the untreated sample (Figure 1). The final consequence of GSK3 inactivation by Akt is the promotion of glucose storage as glycogen, because GS, an enzyme that catalyzes the final step in glycogen synthesis, is a major substrate of GSK3 [25].

**Figure 2:**
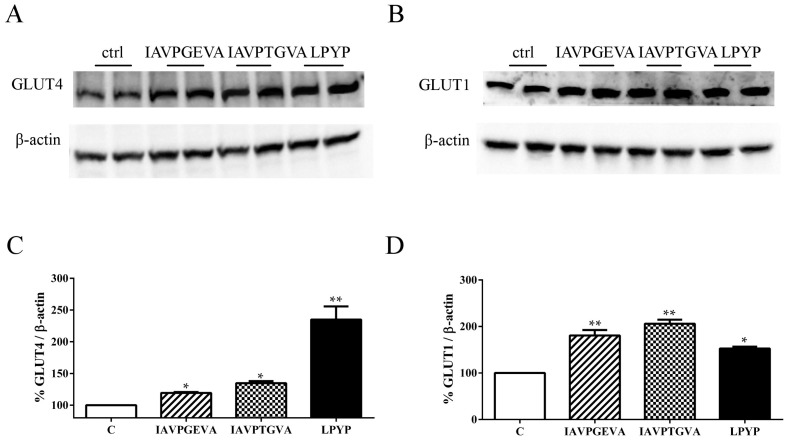
Effect of peptides on glucose transporter 4 (GLUT4) and glucose transporter 1 (GLUT1) protein levels. HepG2 cells (1.5 × 10^5^) were treated with IAVPGEVA (500 μM), IAVPTGVA (500 μM) and LPYP (500 μM) for 24 h. (**A**,**B**) Exemplary western blot analyses of GLUT4 and GLUT1 after treatment. Signals were detected using specific anti-GLUT4, anti-GLUT1 and anti-β-actin primary antibodies (loading untreated sample); (**C**,**D**) Quantitative analyses of immunoblots. GLUT4 and GLUT1 band intensities were quantified by ChemiDoc (BioRad, Hercules, CA, USA) and normalized using β-actin signals. Bars represent averages of duplicate samples ± SEM of seven independent experiments. * *p* < 0.05, ** *p* < 0.001 *versus* untreated sample. C, untreated sample.

As already explained in the introduction, in a previous paper [10] we demonstrated that IAVPGEVA, IAVPTGVA and LPYP activate AMPK through phosphorylation of threonine 172 by 79%, 51% and 100%, respectively, *versus* untreated samples in HepG2 cells. The Akt and AMPK pathway activation suggested investigating the effects of these peptides on the GLUT4 and GLUT1 protein levels, using immunoblot experiments based on specific primary antibodies. Figure 2A–C indicates that IAVPGEVA increased the GLUT4 protein level by 19%, IAVPTGVA by 34% and LPYP by 135%; whereas Figure 2B–D shows that they significantly increased the GLUT1 protein level by 80%, 106% and 52%, respectively, *versus* the untreated sample.

**Figure 3:**
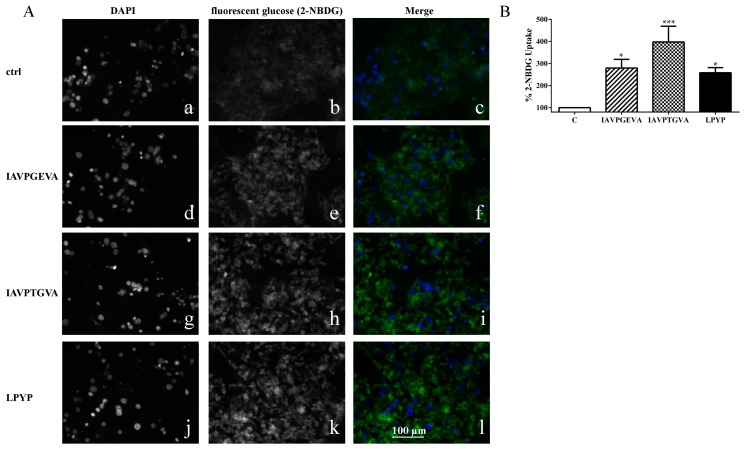
Qualitative and quantitative fluorescent glucose uptake analysis. (**A**) Qualitative uptake. The first column (**a**,**d**,**g**,**j**) represents fluorescence signals of DAPI staining for each peptide. The second column (**b**,**e**,**h**,**k**) represents fluorescent glucose, 2-(*N*-(7-nitrobenz-2-oxa-1,3-diazol-4-yl)amino)-2-deoxyglucose (2-NBDG) signals for each peptide (exciting at 485 nm). The third column (**c**,**f**,**i**,**l**) is the merge of the first and second column (blue signal is DAPI; green signal is glucose staining). Fluorescence images of 20× magnification areas were collected using a Zeiss Axioplan 2 microscope; (**B**) Quantitative analysis of glucose uptake. HepG2 cells (3 × 10^4^) were cultured in complete growth medium, then after 2d treated with IAVPGEVA (50 μM), IAVPTGVA (50 μM) and LPYP (100 μM) for 24 h. The day after, the medium was removed and fluorescent glucose (2-NBDG) (100 μg/mL) was added. After 10 min, excess of 2-NBDG was removed and cells were washed twice with cell based assay buffer. Fluorescent glucose uptake signal was measured by Synergy H1 (Biotek, Winooski, VT, USA). Data points represent averages ± SEM of three independent experiments in triplicate. * *p* < 0.05, *** *p* < 0.0001 *versus* untreated sample. C, untreated sample.

These findings clearly suggest that each soy peptide increases either the GLUT4 or GLUT1 protein levels, but with some differences, since the level of GLUT1, the main isoform responsible for glucose entry into HepG2 cells [16], is enhanced more by IAVPGEVA and IAVPTGVA, whereas the level of GLUT4 is increased much more efficiently by LPYP. Based on these molecular results, qualitative and quantitative experiments were carried out in order to evaluate whether the treatments with these peptides change the functional capability of HepG2 cells to uptake extracellular fluorescent glucose, *i.e.*, 2-(*N*-(7-nitrobenz-2-oxa-1,3-diazol-4-yl)amino)-2-deoxyglucose (2-NBDG). After incubation of HepG2 cells with each peptide for 24 h, the qualitative analysis of glucose uptake by microscopy (Figure 3A) showed that indeed IAVPGEVA, IAVPTGVA and LPYP increase the glucose uptake *versus* untreated sample in HepG2 cells, whereas quantitative data obtained detecting fluorescence signals by a plate reader (Figure 3B) indicated that the 2-NBDG uptake increase is statistically significant. The treatments with 50 μM IAVPGEVA and IAVPTGVA led to a glucose uptake increase by 180% and 298%, respectively, *versus* the untreated sample, whereas after treatment with 100 μM LPYP the glucose uptake was increased by 158% *versus* the untreated sample.

These soy peptides appear to modulate the glucose metabolism and uptake through the activation of Akt and AMPK pathway. In more detail, the activation of Akt, through an increase of phosphorylation at serine 473, leads to the inhibition of GSK3, which in turn produces a positive GS regulation and formation of hepatic glycogen. In parallel, the increased GLUT4 and GLUT1 protein levels lead to an improvement of glucose uptake by HepG2 cells, mainly due to the GLUT1 transporter activity on cellular membranes. Based on this evidence, it seems possible to hypothesize that the ability of these peptides to modulate either glucose or cholesterol metabolism may be due to the synergic activation of Akt and AMPK.

**Figure 4:**
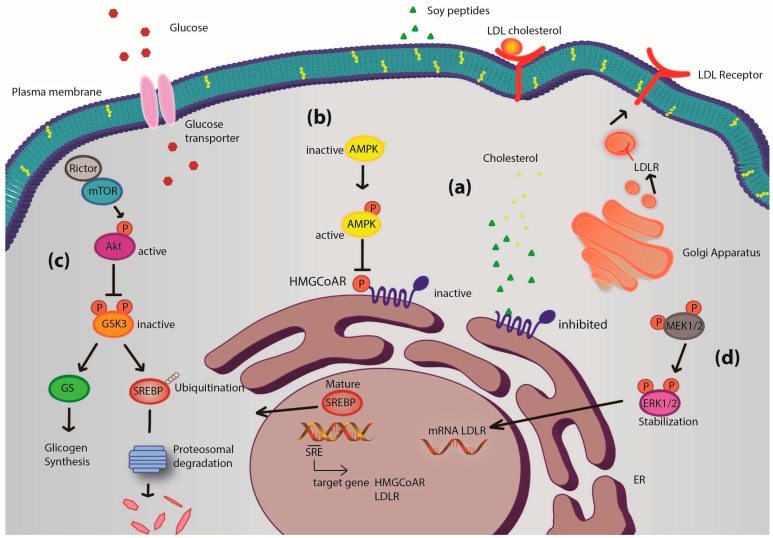
Potential mechanism of action of soy peptides in HepG2 cells. Upon cell penetration, they act as competitive inhibitors of 3-hydroxy-3-methylglutaryl CoA reductase (HMGCoAR) leading to an intracellular cholesterol synthesis reduction. The consequence is the activation of the transcription factor sterol regulatory element-binding proteins (SREBP) 2, which leads to low density lipoprotein receptor (LDLR) and HMGCoAR genes transcription, with subsequent increase of LDLR and HMGCoAR protein levels and LDLR localization in plasma membrane (**a**); In parallel, soy peptides reduce cholesterol production also by activation of the monophosphate-activated protein kinase (AMPK) pathway through an increase of phosphorylation at Thr-172, which in turn inactivates its target substrate HMGCoAR through phosphorylation at serine-872 and produces an increase of the activity of glucose transporters (**b**); IAVPGEVA, IAVPTGVA and LPYP enhance Akt activation, through an increase of phosphorylation at Ser473, which in turn inactivates glycogen synthase kinase 3 αβ (GSK3αβ), a kinase that contributes to proteasomal degradation of SREBP2 [10,28,29]. Moreover, GSK3αβ, inactivated by Akt phosphorylation at Ser21/9, does not inhibit glycogen synthase (its substrate) that can convert the increased up-taken glucose in intracellular glycogen (**c**); Finally, extracellular-signal-regulated kinases (ERK) 1/2 pathway activation [10] could lead to stabilization of mRNA levels of LDLR contributing to increase LDLR protein levels in plasma membrane (**d**). Thus, the distinct modulation of four pathways leads to an increased LDLR activity, which can bind and carry extracellular LDL in HepG2 cells with final hypocholesterolemic effects. Moreover, Akt and AMPK pathway activations are correlated with glucose metabolism modulation, which leads to an increase of glucose-uptake by human hepatic cells.

Figure 4 presents a general picture of the metabolic pathways modulated by these soy peptides in HepG2 cells. Interestingly, the intracellular pathways involving Akt and AMPK are the same involved in the hypoglycemic molecular mechanism of some well-known anti-diabetic drugs, such as metformin [26] and thiazolidinediones [27].

## 3. Experimental Section

### 3.1. Materials

Dulbecco’s modified Eagle’s medium (DMEM), modified Eagle’s medium (MEM), l-glutamine, fetal bovine serum (FBS), phosphate buffered saline (PBS), penicillin/streptomycin, chemiluminescent reagent and 96-well plates were purchased from Euroclone (Milan, Italy). Bovine serum albumin (BSA), RIPA buffer, the antibody against β-actin were bought from Sigma-Aldrich (St. Louis, MO, USA). The antibodies rabbit Ig-HRP, mouse Ig-HRP, PMSF, Na-orthovanadate inhibitors and goat anti-rabbit Ig-HRP were purchased from Santa Cruz Biotechnology Inc. (Santa Cruz, CA, USA). The antibodies against phospho-Akt (Ser473) and GSK3 α/β (Ser21/9) were purchased from Cell Signaling Technologies (Danvers, MA, USA); whereas the antibodies against GLUT1 and GLUT4 were purchased from GeneTex (Irvine, CA, USA). The inhibitor cocktail Complete Midi from Roche (Basel, Switzerland). Mini protean TGX pre-cast gel 7.5% and Mini nitrocellulose Transfer Packs were purchased from BioRad (Hercules, CA, USA). The 2-NBD-glucose (2-NBDG) and cell based assay buffer were purchased from Cayman Chemical Company (Ann Arbor, MI, USA). IAVPGEVA, IAVPTGVA and LPYP were synthesized (>95% purity by HPLC) by PRIMM (Milan, Italy).

### 3.2. Cell Culture

The HepG2 cell line was bought from ATCC (HB-8065, ATCC from LGC Standards, Milan, Italy). It was cultured in high glucose DMEM with stable l-glutamine supplemented with 10% FBS, 100 U/mL penicillin, 100 µg/mL streptomycin (complete growth medium) and incubated at 37 °C under 5% CO_2_ atmosphere. HepG2 cells were used for no more than 20 passages after thawing, because the increase of the number of passages may change the cell characteristics and impair assay results.

### 3.3. Western Blot Analysis

1.5 × 10^5^ HepG2 cells/well (24-well plate) were treated with IAVPGEVA, IAVPTGVA and LPYP peptides (each 500 μM, dissolved in water) or with vehicle (water, untreated sample) for 24 h. At the end of the treatment, cells were scraped in 40 μL ice-cold lysis buffer (RIPA buffer + inhibitor cocktail + 1:100 PMSF + 1:100 Na-orthovanadate) and transferred in an ice-cold microcentrifuge tube. After centrifugation at 13,300 rpm for 15 min at 4 °C, the supernatant was recovered and transferred in a new ice-cold tube. Total proteins were quantified by the Bradford method and 50 μg of total proteins loaded on a pre-cast 7.5% sodium dodecyl sulfate—polyacrylamide (SDS-PAGE) gel at 130 V for 45 min. Subsequently, the gel was pre-equilibrated with 0.04% SDS in H_2_O for 15 min at room temperature (RT) and transferred to a nitrocellulose membrane (Mini nitrocellulose Transfer Packs), using a Trans-blot Turbo at 1.3 A, 25 V for 7 min. Target proteins, on milk blocked membrane, were detected by primary antibodies as follows: rabbit anti-Akt (Ser473), anti-phospho-GSK3αβ (Ser21/Ser9), anti-GLUT1, anti-GLUT4 and anti-β-actin. Secondary antibodies conjugated with HRP and a chemiluminescent reagent were used to visualize target proteins and their signals were quantified using the Image Lab Software (BioRad, Hercules, CA, USA). The internal control β-actin was used to normalize loading variations.

### 3.4. Fluorescent Glucose Uptake Cell Based Assay

3 × 10^4^ HepG2 cells/well were seeded in 96-well plates and kept in complete growth medium for 2 d before treatment. The third day, cells were washed once with PBS and they were treated with 50 µM of IAVPGEVA or IAVAPTGVA and 100 µM of LPYP dissolved in water, respectively, or vehicle (water) in MEM w/o FBS for 24 h. The following day, the medium was removed and 75 μL/well of 2-NBDG (100 μg/mL) in MEM w/o FBS was added for 10 min at 37 °C. Excess of 2-NBDG was aspirated without disrupting the HepG2 layer and cells washed twice with 100 μL of cell based assay buffer. After that, the degree of glucose uptake was measured and quantified using the Synergy H1 fluorescent plate reader from Biotek (excitation and emission wavelengths 485 and 535 nm, respectively). Furthermore, the qualitative glucose uptake was evaluated collecting fluorescent images using Zeiss Axioplan 2 microscope (Oberkochen, Germania). Staining of the nucleus were carried out incubating for 15 min at RT DAPI solution (30 ng/mL). Images were captured at 20× magnification areas.

### 3.5. Statistically Analysis

Statistical analyses were carried out by one-way ANOVA (Graphpad Prism 6 (La Jolla, CA, USA) followed by Dunnett’s test. Values were expressed as means ± SEM; *p*-values < 0.05 were considered to be significant.

## 4. Conclusions

To our knowledge, this is the first report highlighting the activity of some specific peptides from soy glycinin on glucose metabolism and homeostasis in human hepatic cells. Of course, in order to evaluate the final relevance of this study, an open issue is the *in vivo* absorption of these peptides: a very recent paper has provided some positive hints on the absorption of other soy peptides using a Caco2 model system [30]. Natural products are a rich source of leading compounds for drug discovery [31]. In this scenario, these soy peptides might be novel candidates for developing new compounds able to modulate positively cell-signaling pathways. Indeed, recent literature reports other examples of natural-product-like compounds acting as modulator of intracellular cell-signaling pathways [32,33].

## References

[1] Braithwaite M.C., Tyagi C., Tomar L.K., Kumar P., Choonara Y.E., Pillay V. (2014). Nutraceutical-based therapeutics and formulation strategies augmenting their efficiency to complement modern medicine: An overview. J. Funct. Foods.

[2] Chen Z.-Y., Ma K.Y., Liang Y., Peng C., Zuo Y. (2011). Role and classification of cholesterol-lowering functional foods. J. Funct. Foods.

[3] Scicchitano P., Cameli M., Maiello M., Modesti P.A., Muiesan M.L., Novo S., Palmiero P., Saba P.S., Pedrinelli R., Ciccone M.M. (2014). Nutraceuticals and dyslipidaemia: Beyond the common therapeutics. J. Funct. Foods.

[4] Sirtori C.R., Eberini I., Arnoldi A. (2007). Hypocholesterolaemic effects of soya proteins: Results of recent studies are predictable from the Anderson meta-analysis data. Br. J. Nutr..

[5] Harland J., Haffner T. (2008). Systematic review, meta-analysis and regression of randomised controlled trials reporting an association between an intake of circa 25g soya protein per day and blood cholesterol. Atherosclerosis.

[6] Duranti M., Lovati M.R., Dani V., Barbiroli A., Scarafoni A., Castiglioni S., Ponzone C., Morazzoni P. (2004). The α’ subunit from soybean 7S globulin lowers plasma lipids and upregulates liver β-VLDL receptors in rat feed a hypercholesterolemic diet. J. Nutr..

[7] Gianazza E., Eberini I., Arnoldi A., Wait R., Sirtori C.R. (2003). A proteomic investigation of isolated soy proteins with variable effects in experimental and clinical studies. J. Nutr..

[8] Lovati M.R., Manzoni C., Gianazza E., Arnoldi A., Kurowska E., Carroll K.K., Sirtori C.R. (2000). Soy protein peptides regulate cholesterol homeostasis in Hep G2 cells. J. Nutr..

[9] Pak V.V., Koo M., Lee N., Kim M.S., Kwon D.Y. (2005). Structure-activity relationships of the peptide Ile-Ala-Val-Pro and its derivatives revealed using the semi-empirical AM1 method. Chem. Nat. Comp..

[10] Lammi C., Zanoni C., Arnoldi A. (2015). IAVPGEVA, IAVPTGVA, and LPYP, three peptides from soy glycinin modulates cholesterol metabolism in HepG2 cells through the activation of the LDLR-SREBP2 pathway. J. Funct. Foods.

[11] Pak V.V., Koo M.S., Kasymova T.D., Kwon D.Y. (2005). Isolation and identification of peptides from soy 11S-globulin with hypocholesterolemic activity. Chem. Nat. Comp..

[12] Pak V.V., Koo M., Kwon D.Y., Yun L. (2012). Design of a highly potent inhibitory peptide acting as a competitive inhibitor of HMG-CoA reductase. Amino Acids.

[13] Coughlan K.A., Valentine R.J., Ruderman N.B., Saha A.K. (2014). AMPK activation: A therapeutic target for type 2 diabetes?. Diabetes Metab. Syndr. Obes..

[14] Viollet B., Lantier L., Devin-Leclerc J., Hebrard S., Amouyal C., Mounier R., Foretz M., Andreelli F. (2009). Targeting the AMPK pathway for the treatment of Type 2 diabetes. Front. Biosci..

[15] Kerimi A., Jailani F., Williamson G. (2015). Modulation of cellular glucose metabolism in human HepG2 cells by combinations of structurally related flavonoids. Mol. Nutr. Food Res..

[16] Wilkening S., Stahl F., Bader A. (2003). Comparison of primary human hepatocytes and hepatoma cell line HepG2 with regard to their biotransformation properties. Drug Metab. Dispos..

[17] Nakajima K., Yamauchi K., Shigematsu S., Ikeo S., Komatsu M., Aizawa T., Hashizume K. (2000). Selective attenuation of metabolic branch of insulin receptor down-signaling by high glucose in a hepatoma cell line, HepG2 cells. J. Biol. Chem..

[18] Kraniou Y., Cameron-Smith D., Misso M., Collier G., Hargreaves M. (2000). Effects of exercise on GLUT-4 and glycogenin gene expression in human skeletal muscle. J. Appl. Physiol..

[19] Holmes B.F., Kurth-Kraczek E.J., Winder W.W. (1999). Chronic activation of 5′-AMP-activated protein kinase increases GLUT-4, hexokinase, and glycogen in muscle. J. Appl. Physiol..

[20] Ishihara K., Oyaizu S., Fukuchi Y., Mizunoya W., Segawa K., Takahashi M., Mita Y., Fukuya Y., Fushiki T., Yasumoto K. (2003). A soybean peptide isolate diet promotes postprandial carbohydrate oxidation and energy expenditure in type II diabetic mice. J. Nutr..

[21] Oliva M.E., Selenscig D., D’Alessandro M.E., Chicco A., Lombardo Y.B. (2011). Soya protein ameliorates the metabolic abnormalities of dysfunctional adipose tissue of dyslipidaemic rats fed a sucrose-rich diet. Br. J. Nutr..

[22] Anderson J.W., Blake J.E., Turner J., Smith B.M. (1998). Effects of soy protein on renal function and proteinuria in patients with type 2 diabetes. Am. J. Clin. Nutr..

[23] Dove E.R., Mori T.A., Chew G.T., Barden A.E., Woodman R.J., Puddey I.B., Sipsas S., Hodgson J.M. (2011). Lupin and soya reduce glycaemia acutely in type 2 diabetes. Br. J. Nutr..

[24] Roblet C., Doyen A., Amiot J., Pilon G., Marette A., Bazinet L. (2014). Enhancement of glucose uptake in muscular cell by soybean charged peptides isolated by electrodialysis with ultrafiltration membranes (EDUF): Activation of the AMPK pathway. Food Chem..

[25] Cross D.A., Alessi D.R., Cohen P., Andjelkovich M., Hemmings B.A. (1995). Inhibition of glycogen synthase kinase-3 by insulin mediated by protein kinase B. Nature.

[26] Turban S., Stretton C., Drouin O., Green C.J., Watson M.L., Gray A., Ross F., Lantier L., Viollet B., Hardie D.G. (2012). Defining the contribution of AMP-activated protein kinase (AMPK) and protein kinase C (PKC) in regulation of glucose uptake by metformin in skeletal muscle cells. J. Biol. Chem..

[27] Brunmair B., Staniek K., Gras F., Scharf N., Althaym A., Clara R., Roden M., Gnaiger E., Nohl H., Waldhäusl W. (2004). Thiazolidinediones, like metformin, inhibit respiratory complex I: A common mechanism contributing to their antidiabetic actions?. Diabetes.

[28] Lammi C., Zanoni C., Scigliuolo G.M., D’Amato A., Arnoldi A. (2014). Lupin peptides lower low-density lipoprotein (LDL) cholesterol through an up-regulation of the LDL receptor/sterol regulatory element binding protein 2 (SREBP2) pathway at HepG2 cell line. J. Agric. Food Chem.

[29] Krycer J.R., Sharpe L.J., Luu W., Brown A.J. (2010). The Akt-SREBP nexus: Cell signaling meets lipid metabolism. Trends Endocrinol. Metab..

[30] Amigo-Benavent M., Clemente A., Caira S., Stiuso P., Ferranti P., del Castillo M.D. (2014). Use of phytochemomics to evaluate the bioavailability and bioactivity of antioxidant peptides of soybean β-conglycinin. Electrophoresis.

[31] Harvey A.L., Edrada-Ebel R., Quinn R.J. (2015). The re-emergence of natural products for drug discovery in the genomics era. Nat. Rev. Drug Discov..

[32] Liu L.J., Leung K.H., Chan D.S.H., Wang Y.T., Ma D.L., Leung C.H. (2014). Identification of a natural product-like STAT3 dimerization inhibitor by structure-based virtual screening. Cell Death Dis..

[33] Chan D.S., Lee H.M., Yang F., Che C.M., Wong C.C., Abagyan R., Leung C.H., Ma D.L. (2010). Structure-based discovery of natural-product-like TNF-α inhibitors. Angew. Chem. Int. Ed. Eng..

